# Role of Protein Mannosylation in the *Candida tropicalis*-Host Interaction

**DOI:** 10.3389/fmicb.2019.02743

**Published:** 2019-11-28

**Authors:** Marco J. Hernández-Chávez, Diana M. Clavijo-Giraldo, Ádám Novák, Nancy E. Lozoya-Pérez, José A. Martínez-Álvarez, Roberta Salinas-Marín, Nahúm V. Hernández, Iván Martínez-Duncker, Attila Gácser, Héctor M. Mora-Montes

**Affiliations:** ^1^Departamento de Biología, División de Ciencias Naturales y Exactas, Campus Guanajuato, Universidad de Guanajuato, Guanajuato, Mexico; ^2^Department of Microbiology, University of Szeged, Szeged, Hungary; ^3^Laboratorio de Glicobiología Humana y Diagnóstico Molecular, Universidad Autónoma del Estado de Morelos, Cuernavaca, Mexico; ^4^MTA-SZTE “Lendület” Mycobiome Research Group, University of Szeged, Szeged, Hungary

**Keywords:** cell wall, innate immunity, host-fungus interplay, virulence, protein glycosylation

## Abstract

Mannans are components of the fungal wall attached to proteins via *N*- or *O*-linkages. In *Candida albicans*, Och1 is an α1,6-mannosyltransferase that adds the first mannose unit to the *N*-linked mannan outer chain; whereas Pmr1 is an ion pump that imports Mn^2+^ into the Golgi lumen. This cation is the cofactor of Golgi-resident mannosyltransferases, and thus Pmr1 is involved in the synthesis of both *N*- and *O*-linked mannans. Since we currently have limited information about the genetic network behind the *Candida tropicalis* protein mannosylation machinery, we disrupted *OCH1* and *PMR1* in this organism. The *C. tropicalis pmr1*Δ and *och1*Δ mutants showed increased doubling times, aberrant colony and cellular morphology, reduction in the wall mannan content, and increased susceptibility to wall perturbing agents. These changes were accompanied by increased exposure of both β1,3-glucan and chitin at the wall surface of both mutant strains. Our results showed that *O*-linked mannans are dispensable for cytokine production by human mononuclear cells, but *N*-linked mannans and β1,3-glucan are key ligands to trigger cytokine production in a co-stimulatory pathway involving dectin-1 and mannose receptor. Moreover, we found that the *N*-linked mannan core found on the surface of *C. tropicalis och1*Δ null mutant was capable of inducing cytokine production; and that a mannan-independent pathway for IL-10 production is present in the *C. tropicalis*-mononuclear cell interaction. Both mutant strains showed virulence attenuation in the *Galleria mellonella* and the mouse model of systemic candidiasis. Therefore, mannans are relevant for cell wall composition and organization, and for the *C. tropicalis*-host interaction.

## Introduction

Candidiasis, a superficial or deep-seated infection, is caused by members of the *Candida* genus, and thus far *Candida albicans* is the most frequent species isolated from infected tissues. In most of the cases, superficial candidiasis is a benign disease with low morbidity rates; however, systemic candidiasis is a life-threatening condition that is associated with significant rates of morbidity and mortality (Brown et al., 2012). *Candida tropicalis* is among the causative agents of candidiasis that are usually isolated from lesions. This species is commonly found in tropical countries and causes 33–48% of systemic candidiasis (Kothavade et al., 2010; Wang et al., 2016). It is a regular causative agent of candidiasis in neutropenic patients and in recent years has shown increased resistance to antifungal drugs, in particular to fluconazole (Kothavade et al., 2010; Zuza-Alves et al., 2017).

The secreted macromolecules, the capsule, and the cell wall are the fungal components that participate in the early stages of the host-fungus interaction and are key players in the establishment of an immune response against the fungal pathogen. The cell wall of *C. albicans* has been thoroughly characterized and significant amount of information is already available about its role during the interaction with components of the immune system (Díaz-Jiménez et al., 2012; Gow and Hube, 2012; Hall and Gow, 2013; Hall et al., 2013; West et al., 2013; Estrada-Mata et al., 2015; Netea et al., 2015; Erwig and Gow, 2016; Navarro-Arias et al., 2016; Perez-Garcia et al., 2016; Hernández-Chávez et al., 2017; Garcia-Carnero et al., 2018). The *C. albicans* cell wall is composed of chitin, β1,3- and β1,6-glucans that are regarded as structural polysaccharides, localized closer to the plasma membrane, and covered by an outer layer composed of *N*- and *O*-linked mannoproteins (Klis et al., 2001). Thus far, we have evidence indicating that most of the cell wall components are engaged with innate immune receptors that signaling involved in cytokine production and phagocytosis (Martinez-Alvarez et al., 2014). The *N*-linked mannans are recognized by mannose receptor, DC-SIGN, Mincle, dectin-2, and dectin-3 (Netea et al., 2006; Cambi et al., 2008; Wells et al., 2008; Saijo et al., 2010; Zhu et al., 2013); the *O*-linked mannans interact with TLR4 (Netea et al., 2006), β1,3-glucan with dectin-1 and TLR2 (Brown and Gordon, 2001; Netea et al., 2006), and chitin with the mannose receptor, TLR9, and NOD2 (Wagener et al., 2014).

Since genomic analyses have demonstrated that *C. albicans* and *C. tropicalis* are closely related species (Butler et al., 2009), it is assumed the cell wall of both organisms should be similar. So far, it has been reported the presence of chitin, β1,6- and β1,3-glucans, and *N*-linked mannans that contain lateral chains composed of α1,2- and β1,2-mannose units (Kobayashi et al., 1994; Bizerra et al., 2011; Mesa-Arango et al., 2016; Navarro-Arias et al., 2019). Moreover, the proportion of polysaccharides composing the cell wall is similar for both *C. albicans* and *C. tropicalis* (Navarro-Arias et al., 2019). The *N*-linked mannan outer chain is also modified with phosphomannan, a β1,2-oligomannoside attached to the glycan by a mannosyl-phosphate residue (Kobayashi et al., 1994), which is more abundant in the *C. tropicalis* cell wall than in *C. albicans* (Navarro-Arias et al., 2019). In quantitative terms, *C. tropicalis* has a similar amount of cell wall protein than *C. albicans*, but higher wall porosity, suggesting shorter mannan branches (Navarro-Arias et al., 2019). Despite the structure has not been described, the *C. tropicalis* cell wall contains *O*-linked mannans, which are as abundant as those found in *C. albicans* (Navarro-Arias et al., 2019). Even though the cell wall structure of *C. tropicalis* is similar to that described for *C. albicans*, these subtle differences may lead to differential recognition of both pathogens by components of the immune system. *C. tropicalis* induces higher levels of pro- and anti-inflammatory cytokines than *C. albicans* when interacting with human peripheral blood mononuclear cells (PBMCs) (Navarro-Arias et al., 2019), with a strong dependence on dectin-1 engagement with its ligand to induce cytokine production (Duan et al., 2018; Navarro-Arias et al., 2019). In addition, *C. tropicalis* is more readily phagocytosed by human monocyte-derived macrophages, than *C. albicans* cells, in a phosphomannan-dependent mechanism (Hernandez-Chavez et al., 2018; Navarro-Arias et al., 2019). When *C. tropicalis* and *C. albicans* interact with dendritic cells, only the former is capable of inducing the formation of some fungipods (Neumann and Jacobson, 2010). In contrast with our current knowledge in the *C. albicans*-host interaction, the protection against the systemic disease caused by *C. tropicalis* does not require IL-17 signaling but the CARD9-dependent production of TNF-α that enhances the antifungal ability of neutrophils (Whibley et al., 2015).

Besides the importance of the immune cell-*Candida* interaction, mannans are key players in maintaining the cell wall integrity, cellular and colonial morphology, as well as in determining biofilm formation and virulence (Bates et al., 2005, 2006, 2013; Munro et al., 2005; Prill et al., 2005; Mora-Montes et al., 2007, 2010; Hall et al., 2013; West et al., 2013; Estrada-Mata et al., 2015; Navarro-Arias et al., 2016, 2017; Perez-Garcia et al., 2016).

The Golgi-resident P-type ATPase (EC: 7.2.2.10), Pmr1, is an ion pump that imports the mannosyltransferase cofactor Mn^2+^ into the Golgi lumen, allowing proper modification of both *N*- and *O*-linked mannans by Golgi-resident mannosyltransferases (Bates et al., 2005). In *Botrytis cinerea*, *C. albicans* and *C. guilliermondii*, disruption of *PMR1* affected the cell wall composition and proper elongation of both *N*- and *O*-linked mannans (Bates et al., 2005; Plaza et al., 2015; Navarro-Arias et al., 2016) and thus, the *Candida* null mutants stimulated poor cytokine production by human PBMCs and dendritic cells, reduced uptake by macrophages, and showed virulence attenuation (Netea et al., 2006; Cambi et al., 2008; McKenzie et al., 2010; Navarro-Arias et al., 2016).

The *OCH1* encodes a Golgi-resident α1,6-mannosyl- transferase (EC: 2.4.1.232) that primes the elaboration of the *N-*linked mannan outer chain (Martinez-Duncker et al., 2014). Loss of this gene in both *C. albicans* and *C. parapsilosis* increased the sensitivity to cell wall perturbing agents, affected the cell wall composition, the ability to stimulate cytokine production by human PMBCs and dendritic cells, and the uptake by macrophages (Bates et al., 2006; Netea et al., 2006; Cambi et al., 2008; McKenzie et al., 2010; Perez-Garcia et al., 2016). Similar to the *pmr1*Δ null mutants, the *och1*Δ mutants showed virulence attenuation, but only defects in the structure of *N*-linked mannans, with normal *O*-linked mannans decorating the cell surface (Bates et al., 2006; Perez-Garcia et al., 2016). Besides the genetic approach to assess the relevance of mannans in the fungal biology, the chemical removal of *O*-linked mannans by β-elimination or the enzymatic trimming of *N*-linked mannans with endoglycosidase H (endo H) have positively impacted in our current knowledge about the versatile functions of mannans in the biology of *Candida* spp. and other fungal species (Hamada et al., 1981; Hazen and Glee, 1994; Mormeneo et al., 1994; Goins and Cutler, 2000; Spreghini et al., 2003; Navarro-Arias et al., 2016, 2017, 2019; Perez-Garcia et al., 2016; Martinez-Alvarez et al., 2017; Lozoya-Perez et al., 2019).

Here, to assess the relevance of mannans in the biology of *C. tropicalis*, we generated single null mutants in the genes *OCH1* and *PMR1* and conducted the phenotypical characterization with an emphasis on the cell wall composition and status of the protein glycosylation pathways. In addition, the ability to stimulate cytokine production by human PBMCs, and the virulence in both mouse and *Galleria mellonella* models were evaluated.

## Results

### Identification and Disruption of *Candida tropicalis PMR1* and *OCH1*

The *C. tropicalis PMR1* and *OCH1* sequences were identified following a standard BLAST analysis at the NCBI website^1^, using the protein sequences of *C. albicans* Pmr1 (GenBank accession code XP_720380) and Och1 (GenBank accession code AOW28617) as a query. The putative ortholog of *C. albicans* Pmr1 was the product encoded by the locus EER31186 (GenBank accession code EER31186). The open reading frame (ORF) spans 2760 bp with no putative introns identified and is predicted to encode a polypeptide of 919 amino acids, with 88 and 95% identity and similarity to *C. albicans* Pmr1, respectively. The putative protein is predicted to fold in nine transmembrane domains, to contain the canonical motif ^352^DKTGTLT that includes the aspartic acid involved in the phosphorylation of P-type ATPases (Lutsenko and Kaplan, 1995; Bates et al., 2005), the type A and C cation transport domains, and a putative dehalogenase domain; all these traits already identified in the *C. albicans* Pmr1 (Bates et al., 2005). For the case of *OCH1*, the putative ortholog of *C. albicans* Och1 was the gene product of the locus EER33436 (GenBank accession code EER33436), whose ORF spans 1131 bp with no intron predicted. The encoded protein spans 376 amino acids, with 72 and 85% identity and similarity to *C. albicans* Och1, respectively, is predicted to be a type-II transmembrane protein belonging to the glycosyltransferase family 32, and contains the sequence ^180^DXD, a common signature sequence of proteins that use divalent cations, such as Mn^2+^ as a cofactor during the transference of monosaccharides from a nucleotide-activated sugar to oligo and polysaccharides (Bates et al., 2006). To test these hypotheses generated by bioinformatic means, we next complemented *C. albicans pmr1*Δ and *och1*Δ null mutants (Bates et al., 2005, 2006) with the corresponding *C. tropicalis* ORFs under the control of the strong *C. albicans ACT1* promoter (Barelle et al., 2004). As previously reported (Bates et al., 2005, 2006), the *C. albicans pmr1*Δ and *och1*Δ null mutants showed defects in the cell wall composition, with low mannan and phosphomannan content and increased glucan levels (Table 1). Chitin content was only modified in the *och1*Δ null mutant but not in *pmr1*Δ cells (Table 1). Moreover, in line with the reduction in mannan content, both mutant strains showed increased cell wall porosity (Table 1). Expression of *C. tropicalis PMR1* or *OCH1* in the corresponding *C. albicans* null mutant restored this phenotype to levels comparable to those found in the wild-type (WT) control strain (Table 1). Collectively, these data suggest that *C. tropicalis PMR1* and *OCH1* are the functional orthologs of *C. albicans PMR1* and *OCH1*, respectively.

**TABLE 1:** Complementation of *Candida albicans pmr1*Δ and *och1*Δ null mutants with the functional orthologs of *Candida tropicalis.*

	**Cell wall abundance**			**Phosphomannan content (μg)^a^**	**Porosity (%)^b^**
**Strain**	**Chitin (%)**	**Mannan (%)**	**Glucan (%)**		
WT (NGY152)	3.1 ± 1.0	33.7 ± 1.2	63.2 ± 1.8	116.3 ± 9.4	18.1 ± 6.1
*pmr1*Δ (NGY355)	2.9 ± 1.4	6.9 ± 3.0^∗^	90.2 ± 1.3^∗^	30.6 ± 13.5^∗^	80.4 ± 17.9^∗^
*pmr1*Δ + Ct*PMR1* (HMY185)	3.0 ± 1.2	31.8 ± 1.9	65.2 ± 2.5	109.5 ± 13.6	25.5 ± 9.5
*och1*Δ (NGY 357)	5.9 ± 1.0^∗^	10.3 ± 2.2^∗^	83.8 ± 3.1^∗^	39.5 ± 9.4^∗^	43.8 ± 9.4^∗^
*och1*Δ + Ct*OCH1* (HMY148)	2.5 ± 1.3	32.5 ± 1.0	65.0 ± 2.4	106.4 ± 15.9	22.8 ± 8.7

*^*a*^μg of Alcian Blue bound/OD_600_ = 1, ^*b*^Relative to DEAE-Dextran, ^∗^*P* < 0.05, when compared to the WT or complemented strains.*

Next, to generate a *C. tropicalis* strain lacking either *PMR1* or *OCH1* the *SATI* flipper methodology was used to disrupt both alleles of these two genes. The disruption cassettes were constructed in the pSFS2 plasmid (Reuß et al., 2004) and contained 1500 bp regions upstream and downstream the targeted ORF for homologous recombination. For both genes, disruption of the first allele was confirmed by PCR, cells underwent marker recycling, used in the second round of transformation with the same disruption cassette, and again transformation marker recycling, generating the *pmr1*Δ and the *och1*Δ null mutants (Table 2). To generate reintegrant control strains, either *PMR1* or *OCH1* ORF plus regulatory regions were cloned into pSFS2 and this construction used to transform the *pmr1*Δ or *och1*Δ null mutant, respectively. The integration of constructions into the targeted loci was confirmed by PCR (data not shown).

**TABLE 2:** Strains used in this work.

**Strain**	**Organism**	**Origin**	**Genotype**	**References**
MYA-3404	*C. tropicalis*	ATCC	Wild type	ATCC
HMY206	*C. tropicalis*	Derived from MYA-3404	As ATCC MYA-3404, but *pmr1*Δ:*sat1*/*PMR1*	This work
HMY207	*C. tropicalis*	Derived from HMY206	As ATCC MYA-3404, but *pmr1*Δ*:sat1/pmr1*Δ*:sat1*	This work
HMY208	*C. tropicalis*	Derived from HMY207	As ATCC MYA-3404, but *pmr1*Δ*:sat1/pmr1*Δ*:sat1-PMR1*	This work
HMY179	*C. tropicalis*	Derived from MYA-3404	As ATCC MYA-3404, but *och1*Δ:*sat1*/*OCH1*	This work
HMY181	*C. tropicalis*	Derived from HMY179	As ATCC MYA-3404, but *och1*Δ:*sat1*/*och1*Δ:*sat1*	This work
HMY205	*C. tropicalis*	Derived from HMY181	As ATCC MYA-3404, but *och1*Δ:*sat1*/*och1*Δ:*sat1-OCH1*	This work
NGY152	*C. albicans*	Derived from CAI-4	*ura3*Δ-*iro1*Δ:*imm434*/*ura3*Δ-*iro1*Δ:*imm434*; *RPSI*/*rps1*Δ:CIp10	Brand et al., 2004
NGY98	*C. albicans*	Derived from NGY97	*ura3*Δ-*iro1*Δ:*imm434*/*ura3*Δ-*iro1*Δ:*imm434*; *pmr1*Δ:*hisG*/*pmr1*Δ:*hisG*	Bates et al., 2005
NGY355	*C. albicans*	Derived from NGY98	As NGY98, but *RPSI*/*rps1*Δ:CIp10	Bates et al., 2005
HMY185	*C. albicans*	Derived from NGY98	As NGY98, but *RPSI*/*rps1*Δ:p*ACT1*-Ct*PMR1*	This work
NGY205	*C. albicans*	Derived from NGY204	*ura3*Δ-*iro1*Δ:*imm434*/*ura3*Δ-*iro1*Δ:*imm434*; *och1*Δ:*hisG*/*och1*Δ:*hisG*	Bates et al., 2006
NGY357	*C. albicans*	Derived from NGY205	As NGY205, but *RPSI*/*rps1*Δ:CIp10	Bates et al., 2006
HMY148	*C. albicans*	Derived from NGY205	As NGY205, but *RPSI*/*rps1*Δ:p*ACT1*-Ct*OCH1*	This work

**FIGURE 1:**
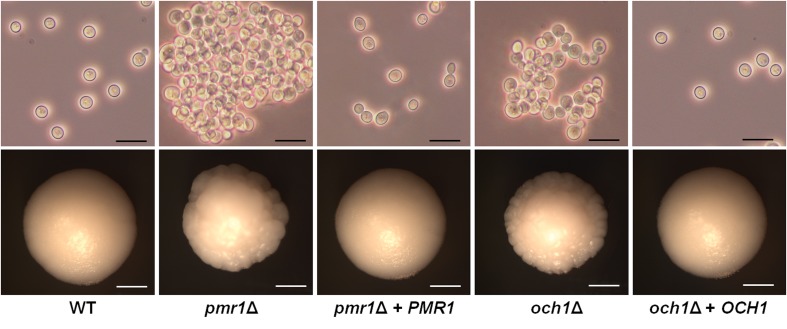
Cell and colony morphology of *Candida tropicalis pmr1*Δ and *och1*Δ null mutants. The upper panels show the cell morphology after growth at 28°C for 15 h in YPD medium, demonstrating cell aggregates in both null mutant strains. Scale bar, 10 μm. The lower panels show colony morphology after 3 days of growth at 28°C on YPD plates. Both null mutants formed colonies with irregular borders and a wrinkled surface. Scale bar, 1 mm. Strains used are MYA-3404 (WT), HMY207 (*pmr1*Δ), HMY208 (*pmr1*Δ + *PMR1*), HMY181 (*och1*Δ), and HMY205 (*och1*Δ + *OCH1*).

Both the *pmr1*Δ and *och1*Δ null mutants showed defects in the cell morphology, forming cell aggregates, which were more prominent in the former (Figure 1). The reintegration of one allele of the disrupted gene reverted the phenotype of the null mutant strains, with a cell morphology similar to that displayed by the WT control strain (Figure 1). In addition, both mutant strains showed defects in the colony morphology when grown on a solid culture medium, forming colonies with irregular borders and a wrinkled surface (Figure 1). Again, the reintegrant control strains showed a phenotype similar to the WT strain (Figure 1). These changes were accompanied by changes in the doubling time at 28°C, with the null mutant strains tending to grow slower than the WT and reintegrant control strains (doubling time of the WT control strain 1.49 ± 0.19 h, *pmr1*Δ strain 2.25 ± 0.24 h, *pmr1*Δ + *PMR1* strain 1.47 ± 0.25 h, *och1*Δ strain 2.22 ± 0.27 h, and *och1*Δ + *OCH1* strain 1.52 ± 0.12 h, *p* < 0.05 when compared the null mutant strain with the WT strain or the corresponding reintegrant strain), and both the *pmr1*Δ and *och1*Δ null mutants, but not the WT or reintegrant control strains, were unable to grow at 42°C (data not shown).

### The *Candida tropicalis pmr1*Δ and *och1*Δ Null Mutants Have Defects in the Cell Wall and Protein Glycosylation Pathways

**FIGURE 2:**
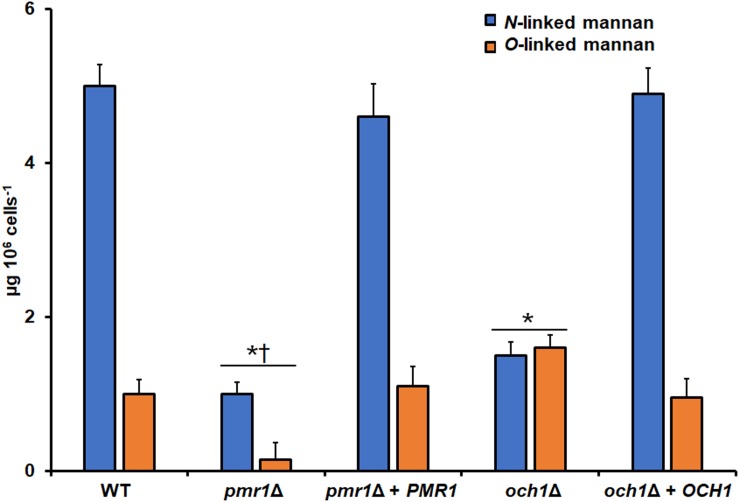
Cell wall mannan content in the *Candida tropicalis pmr1*Δ and *och1*Δ null mutants. Cells were treated with endoglycosidase H or β-elimination to remove *N*-linked mannans or *O*-linked mannans, respectively, the trimmed oligosaccharides were concentrated and the carbohydrate content was quantified as described in methods. Data are means ± SD of three independent experiments performed in duplicates. Strains used are MYA-3404 (WT), HMY207 (*pmr1*Δ), HMY208 (*pmr1*Δ + *PMR1*), HMY181 (*och1*Δ), and HMY205 (*och1*Δ + *OCH1*). ^∗^*P* < 0.05, when compared with either the WT or reintegrant control cells. †*P* < 0.05, when compared with the *och1*Δ null mutant.

**FIGURE 3:**
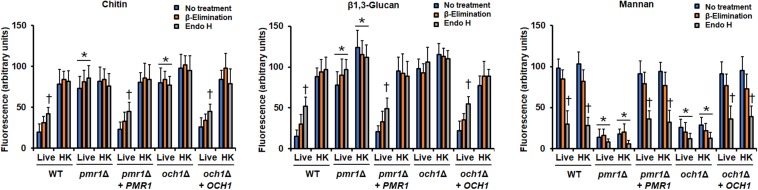
Fluorescent labeling of cell wall chitin, β1,3-glucan, and mannan in the *Candida tropicalis pmr1*Δ and *och1*Δ null mutants. Live or heat-killed (HK) yeast cells were labeled with either fluorescein isothiocyanate-wheat germ agglutinin conjugate that binds chitin, IgG Fc-Dectin-1 chimera that binds β1,3-glucan, or fluorescein isothiocyanate-concanavalin A conjugate that binds mannan, as described in the methods, inspected under fluorescence microscopy, and the fluorescence associated to 300 individual cells recorded. In addition, cells were previously β-eliminated or treated with endoglycosidase H (endo H) before labeling with lectins. ^∗^*P* < 0.05, when compared with live and HK cells from the WT strain. †*P* < 0.05, when compared with no treated cells. Strains used are MYA-3404 (WT), HMY207 (*pmr1*Δ), HMY208 (*pmr1*Δ + *PMR1*), HMY181 (*och1*Δ), and HMY205 (*och1*Δ + *OCH1*).

To assess the role of either *PMR1* or *OCH1* in the *C. tropicalis* wall composition, cell walls were isolated, cleaned, acid-hydrolyzed, and analyzed by High-Performance Anion-Exchange Chromatography with Pulsed Amperometric Detection (HPAEC-PAD). The cell wall composition of the WT control strain was similar to that recently reported for *C. tropicalis*, with a large amount of glucan, followed by mannan, and a small proportion of chitin (Hernandez-Chavez et al., 2018; Table 3). The *pmr1*Δ null mutant showed about 10-fold more chitin content than the WT control strain, a significant 5-fold reduction in the mannan levels, and no changes in the glucan content (Table 3). The *och1*Δ null mutant showed a 3-fold reduction in the mannan content, no changes in the chitin levels and a significant 1.3-fold increment in the glucan content (Table 3). Interestingly, the changes in the content of these three cell wall components were different when both null mutants were compared (Table 3). In both cases, the reintegrant control strains restored the content of these wall components to levels similar to those found in the WT control strain (Table 3). The phosphomannan content and the porosity to DEAE-dextran are wall parameters that have been correlated with defects in the protein glycosylation pathways (Mora-Montes et al., 2007, 2010; Cheng et al., 2011; Navarro-Arias et al., 2016, 2017; Perez-Garcia et al., 2016; Lozoya-Perez et al., 2019). The phosphomannan content was reduced in both null mutants, and this was accompanied by an increment in the cell wall porosity (Table 3). Again, both reintegrant strains showed a phenotype similar to the WT strain (Table 3). To further explore defects in the mannosylation pathways in either the *pmr1*Δ or the *och1*Δ null mutant, cells were subjected to treatment with endo H or β-elimination to remove *N*-linked or *O*-linked mannans, respectively (Navarro-Arias et al., 2016, 2019; Lozoya-Perez et al., 2019). The *N*-linked and *O*-linked mannan content in the WT control strain was similar to that recently reported (Navarro-Arias et al., 2019), with most of the cell wall mannan bound to proteins by *N*-linkages (Figure 2). The *pmr1*Δ null mutant showed a low level of both mannans, whereas the *och1*Δ null mutant had a reduction of *N*-linked mannans and an increment in the *O*-linked mannan content (Figure 2). The mannan content was different when both null mutant strains were compared (Figure 2). The reintegrant control strains displayed a phenotype similar to the WT strain, confirming that the defects found in the mannosylation pathways are associated with the disruption of the genes under analysis (Figure 2). To further confirm the decreased content of cell wall mannan in both mutants, we labeled these oligosaccharides with fluorescein isothiocyanate-concanavalin A conjugate (Con A-FITC) and inspected the cells under fluorescent microscopy. The lectin strongly bound to the mannan of the WT cells, but a weak interaction of this with the cell wall of either the *pmr1*Δ or the *och1*Δ null mutant was observed, confirming of findings of decreased mannan content in these mutants (Figure 3 and Supplementary Figure S1). The reintegrant control strains showed a similar ability to bind Con A-FITC than the WT cells (Figure 3 and Supplementary Figure S1). Since heat inactivation of cells artificially exposes β1,3-glucan and chitin at the cell wall surface (Gow et al., 2007; Mora-Montes et al., 2011; Estrada-Mata et al., 2015; Navarro-Arias et al., 2016, 2017, 2019; Perez-Garcia et al., 2016; Hernandez-Chavez et al., 2018); we next repeated the experiment but using heat-killed (HK) cells for fluorescent labeling. This cell treatment did not have an impact on the ability of Con A-FITC to bind the cell wall (Figure 3 and Supplementary Figure S1). When these experiments were performed with β-eliminated cells we observed a small reduction in the ability of the lectin to bind cell walls, but this was not significant (*P* > 0.05; Figure 3). When endo H-treated cells were used for interaction with Con A-FITC there was a significant reduction in the binding of the lectin by both the live and HK WT and reintegrant control cells (Figure 3). In the case of both mutants, the treatment with endo H decreased the binding of Con A-FITC, but this was not significant (Figure 3).

**TABLE 3:** Cell wall composition of *Candida tropicalis* WT, *pmr1*Δ, *och1*Δ, and reintegrant control strains.

	**Cell wall abundance**			**Phosphomannan content (μg)^a^**	**Porosity (%)^b^**
**Strain**	**Chitin (%)**	**Mannan (%)**	**Glucan (%)**		
WT (MYA-3404)	2.8 ± 1.4	34.0 ± 1.9	63.2 ± 2.2	94.8 ± 6.5	51.5 ± 4.7
*pmr1*Δ (HMY207)	30.2 ± 3.4^*†^	6.7 ± 2.4^*†^	63.1 ± 1.8†	16.9 ± 4.2^*†^	92.9 ± 5.8^∗^
*pmr1*Δ + *PMR1* (HMY208)	3.0 ± 1.2	31.8 ± 2.2	65.2 ± 2.5	91.1 ± 8.8	51.9 ± 9.7
*och1*Δ (HMY181)	3.1 ± 1.3	11.9 ± 1.6^∗^	85.0 ± 2.5^∗^	36.2 ± 12.4^∗^	88.8 ± 2.7^∗^
*och1*Δ + *OCH1* (HMY205)	3.0 ± 1.1	32.1 ± 2.0	64.9 ± 1.9	98.2 ± 7.7	56.9 ± 10.4

*^*a*^μg of Alcian Blue bound/OD_600_ = 1, ^*b*^Relative to DEAE-Dextran, ^∗^*P* < 0.05, when compared to the WT or complemented strains, ^†^*P* < 0.05, when compared to *och1*Δ null mutant.*

Next, we analyzed the organization of the structural polysaccharides chitin and β1,3-glucan within the cell wall, using fluorescence labeling with fluorescein isothiocyanate-wheat germ agglutinin conjugate (WGA-FITC) and an IgG Fc-Dectin-1 chimera, respectively (Graham et al., 2006; Mora-Montes et al., 2011; Marakalala et al., 2013; Estrada-Mata et al., 2015; Navarro-Arias et al., 2016, 2017, 2019; Hernandez-Chavez et al., 2018). Both lectins barely bound to chitin and β1,3-glucan localized in the cell wall of the WT and the re-integrant control strains (Figure 3 and Supplementary Figures S2, S3). Cells from either the *pmr1*Δ or the *och1*Δ null mutant were significantly more labeled than the WT or reintegrant control cells with both lectins, suggesting exposure of chitin and β1,3-glucan at the cell wall surface (Figure 3 and Supplementary Figures S2, S3). The HK WT and reintegrant control cells showed an increased ability to bind both lectins (Figure 3 and Supplementary Figures S2, S3), suggesting that the modest interaction between the lectins and live cells was due to inaccessibility of lectins to polysaccharides, i.e., these are in the inner part of the cell wall. The chitin labeling in both the *pmr1*Δ and *och1*Δ null mutants was similar to that observed in live cells, and β1,3-glucan labeling was increased upon heat inactivation (Figure 3). As in the case of mannan labeling, the β-elimination treatment did not affect the chitin or β1,3-glucan labeling (Figure 3). Upon incubation with endo H, the lectin binding to both polysaccharides from live and HK null mutant strains was similar to the untreated cells, but an increased binding to both chitin and β1,3-glucan was observed in the walls of live WT and reintegrant control cells (Figure 3). Collectively these data confirm that both polysaccharides are exposed at the cell surface of the *pmr1*Δ and *och1*Δ null mutants. In addition, they are in line with the increment in glucan content quantified by HPAEC-PAD.

**FIGURE 4:**
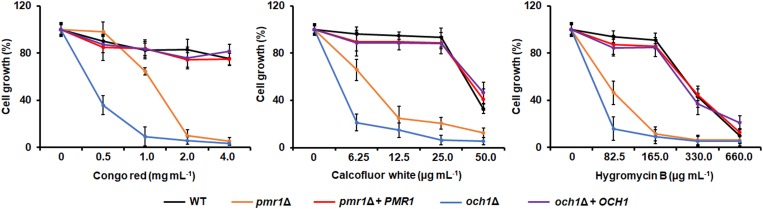
Susceptibility to cell wall perturbing agents in the *Candida tropicalis pmr1*Δ and *och1*Δ null mutants. Yeast cells were incubated in YPD broth supplemented with different concentrations of Congo red, Calcofluor white, or Hygromycin B, and cell growth was determined after incubation for 24 h at 30°C. For normalization, growth results are shown as a percentage of those obtained with the same strain grown in the absence of any perturbing agent. Data are means ± SD of three independent experiments performed in duplicates. Both null mutant strains showed increased susceptibility to the three cell wall perturbing agents analyzed, and were significantly different to either the WT or the reintegrant control strain (*P* < 0.01 when compared by two-way ANOVA). Moreover, the increased susceptibility of the null mutants was different when compared to each other (*P* < 0.05 when compared by two-way ANOVA). Strains used are MYA-3404 (WT), HMY207 (*pmr1*Δ), HMY208 (*pmr1*Δ + *PMR1*), HMY181 (*och1*Δ), and HMY205 (*och1*Δ + *OCH1*).

It has been previously reported that *Candida* mutants in either the *PMR1* or the *OCH1* genes have increased susceptibilities to cell wall perturbing agents (Bates et al., 2005, 2006; Navarro-Arias et al., 2016; Perez-Garcia et al., 2016). Therefore, we analyzed the effect of this kind of compounds on the growth of the *C. tropicalis pmr1*Δ and *och1*Δ null mutants. Congo red, a compound that disturbs the glucan within the cell wall (Kopecka and Gabriel, 1992), had a modest effect on the growth of the WT and reintegrant control strains but both the *pmr1*Δ and *och1*Δ null mutants showed increased susceptibility to this stressor (Figure 4). The WT and reintegrant control strains showed a dose-dependent increased susceptibility to both Calcofluor white and hygromycin B, and this effect was more prominent in the presence of the latter (Figure 4). Calcofluor white binds to chitin; while increased susceptibility to hygromycin B has been reported in yeast cells with defects in the protein glycosylation pathways (Dean, 1995; Mora-Montes et al., 2011). Again, the *pmr1*Δ and *och1*Δ null mutants had increased susceptibility to these perturbing agents (Figure 4). Interestingly, when compared to the ability of both mutants to grow in presence of these three cell wall perturbing agents, the *och1*Δ null mutant was more affected than the strain were *PMR1* was disrupted (*P* < 0.05 in all cases; Figure 4). Altogether, these data indicate that loss of either *PMR1* or *OCH1* affects the *C. tropicalis* protein glycosylation and the cell wall composition, organization, and fitness.

### Removal of Cell Wall Mannans Affects the Ability of *Candida tropicalis* to Stimulate Cytokine Production by Human PBMCs

**FIGURE 5:**
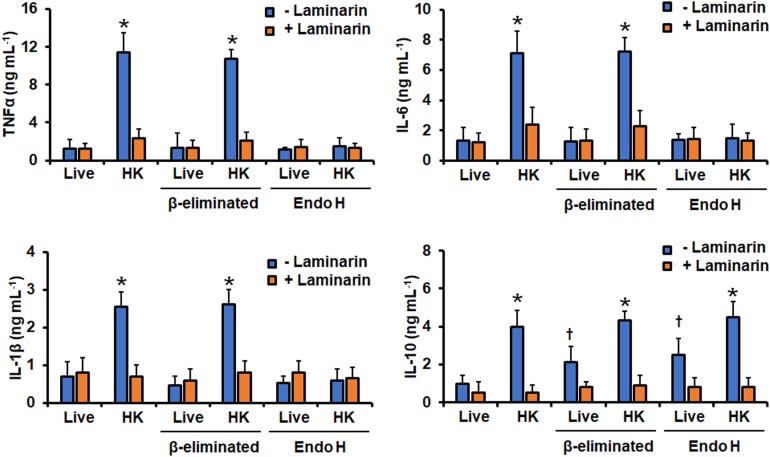
Cytokine production by human PBMCs stimulated with *Candida tropicalis*. Yeast cells and human PBMCs were co-incubated for 24 h, the supernatant saved and used to quantify the levels of secreted TNFα, IL-6, IL-1β, and IL-10. Results (means ± SD) were obtained using samples from seven donors, each assayed in duplicate wells, and the wild type strain MYA-3404. ^∗^*P* < 0.05, when compared with live cells under the same treatment; ^†^*P* < 0.05, when compared with live cells with no treatment. HK, heat-inactivated cells; Endo-H, endoglycosidase H.

Next, we assessed the importance of cell wall mannan on the *C. tropicalis*-host interaction, analyzing the ability of this fungal species to stimulate cytokine production by human PBMCs. We first compared the cytokine profile induced by the WT strain treated with endo H or β-elimination, to remove *N*-linked or *O*-linked mannans, respectively (Navarro-Arias et al., 2016; Perez-Garcia et al., 2016). Moreover, since in other *Candida* species β1,3-glucan is one of the major fungal ligands to stimulate cytokine production and is normally buried behind the mannan layer, and therefore inaccessible to engage with dectin-1 (Gow et al., 2007; Estrada-Mata et al., 2015; Navarro-Arias et al., 2016, 2017, 2019; Perez-Garcia et al., 2016), we also compared the ability of live and HK cells to stimulate cytokine production. Live cells stimulated low levels of TNFα, IL-1β, IL-6, and IL-10, but PMBCs interacting with HK cells produced a significantly higher level of these cytokines (Figure 5). The experimental setting where PBMCs were pre-incubated with laminarin and challenged with HK cells stimulated cytokine levels similar to those with the live cells (Figure 5). Thus, the high cytokine levels induced by HK cells were associated with the engagement of dectin-1 with its ligand, as reported for other fungal cells (Estrada-Mata et al., 2015; Navarro-Arias et al., 2016, 2017, 2019; Perez-Garcia et al., 2016). Removal of *O*-linked mannans via β-elimination did not affect the TNFα, IL-1β, or IL-6 profiles, but live cells with no *O*-linked mannans on the surface tended to simulate higher IL-10 levels than untreated cells (Figure 5). The trimming of *N*-linked mannans with endo H only affected the ability of live cells to stimulate IL-10, which was higher, when compared with the levels stimulated with untreated cells (Figure 5). The endo H-treated HK cells were not able to stimulate a strong production of TNFα, IL-1β, or IL-6; while the levels of IL-10 were not affected when compared with those stimulated with the untreated HK cells (Figure 5). It is possible to hypothesize that treatment with endo-H could expose cell wall proteins to the extracellular compartment, promoting detachment of these macromolecules from the wall, accounting for the changes in the ability to stimulate cytokine production. However, these is unlikely, as the wall protein content of cells was not affected upon treatment with endo-H (132.4 ± 21.4, 144.1 ± 17.2, 122.9 ± 13.9, and 136.8 ± 15.6 μg of protein mg of cell wall^–1^ for live, live + endo H-, HK, HK + endo H-treated cells, respectively).

**FIGURE 6:**
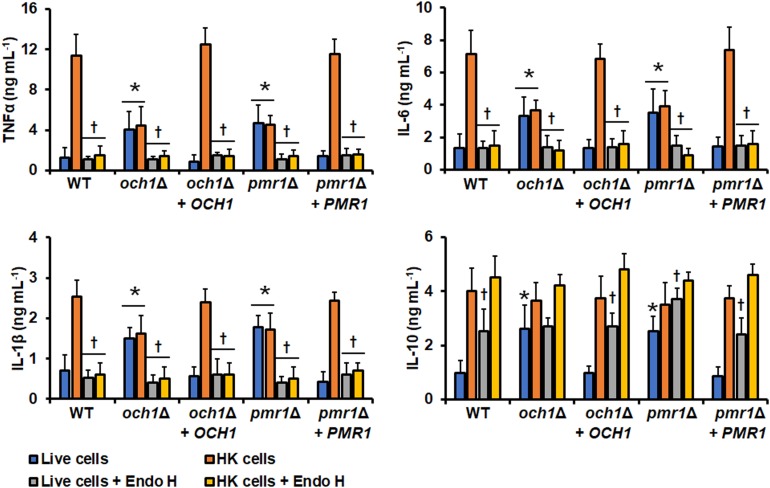
Cytokine production by human PBMCs stimulated with *Candida tropicalis och1*Δ and *pmr1*Δ null mutants. Yeast cells and human PBMC were co-incubated for 24 h, the supernatant saved and used to quantify the levels of secreted TNFα, Il-6, IL-1β, and IL-10. Results (means ± SD) were obtained using samples from seven donors, each assayed in duplicate wells. ^∗^*P* < 0.05, when compared with WT control cells under the same treatment; ^†^*P* < 0.05, when compared with cells without treatment with endo H. HK, heat-inactivated cells; Endo-H, endoglycosidase H. Strains used are MYA-3404 (WT), HMY207 (*pmr1*Δ), HMY208 (*pmr1*Δ + *PMR1*), HMY181 (*och1*Δ), and HMY205 (*och1*Δ + *OCH1*).

**FIGURE 7:**
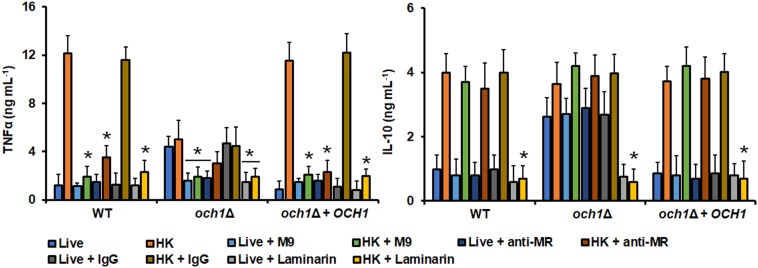
Cytokine production by human PBMCs preincubated with M9, anti-MR, or laminarin and stimulated with the *Candida tropicalis och1*Δ null mutant. human PBMCs were pre-incubated with M9, anti-MR, or laminarin as described in the Materials and Methods section and then stimulated with either live or heat-killed (HK) yeast cells for 24 h. The supernatant of the interactions was saved and used to quantify cytokine production by ELISA. In the experiments using anti-MR antibodies, control assays with PBMCs pre-incubated with an irrelevant IgG were included as a control. Results (means ± SD) were obtained using samples from seven donors, each assayed in duplicate wells. ^∗^*P* < 0.05, when compared with the corresponding live or HK untreated cells. M9, Man_9_GlcNAc_2_; MR, mannose receptor. Strains used are MYA-3404 (WT), HMY181 (*och1*Δ), and HMY205 (*och1*Δ + *OCH1*).

In similar experiments, the ability of the *pmr1*Δ and *och1*Δ null mutant to stimulate cytokine production by human PBMCs was also assessed. Live cells of either the *pmr1*Δ or *och1*Δ null mutant stimulated higher TNFα, IL-6, IL-1β, and IL-10 when compared with the WT live cells (Figure 6). For the three pro-inflammatory cytokines quantified, the stimulated levels using HK mutant cells were similar to those observed in the system with live cells and not sufficient enough to be comparable to those stimulated with the WT HK cells (Figure 6). For IL-10, the cytokine level stimulated with HK cells was comparable with that observed with live mutant cells, but slightly higher, and therefore statistically similar to the levels stimulated with the WT HK cells (*P* = 0.627 when WT and *och1*Δ null mutant cells are compared; *P* = 0.295 when WT and *pmr1*Δ null mutant cells are compared). Both live and HK cells of the reintegrant control strains for *OCH1* and *PMR1* showed an ability to stimulate cytokine production similar to WT control cells (Figure 6). It is worthy of note that it was expected that the cytokine profile stimulated with endo-H-treated WT cells was similar to that observed with cells lacking *OCH1* but results from Figures 5, 6 clearly showed this was not the case. Since Och1 is a protein localized within the Golgi complex, it is expected that the *N*-linked mannan core is properly assembled and transferred to glycoproteins, as demonstrated in *C. albicans* (Bates et al., 2006). Therefore, the cell surface of an *och1*Δ null mutant strain will display the *N*-linked mannan core on the wall surface, which contrasts with the surface of endo-H-treated cells where all the *N*-linked mannan, including the oligosaccharide core, is absent. Thus, we next compared the ability to stimulate cytokine production of the null mutants subjected to endo H treatment. For both live and HK cells treated with endo H, the levels of the proinflammatory cytokines TNFα, IL-6, and IL-1β were similar to those produced in the system where live untreated cells were included (Figure 6). For IL-10 stimulation, the *och1*Δ null mutant did not show any difference in the ability to stimulate this cytokine when compared to the system where endo H was not included (Figure 6). In the case of the *pmr1*Δ null mutant, live cells treated with endo H stimulated significantly higher levels of this cytokine, when compared to the system that not included the glycosidase (Figure 6). The reintegrant control strains for both genes showed a similar ability to stimulate cytokine production to that observed with the WT control cells (Figure 6). To further explore the differences between endo-H-treated WT cells and the *och1*Δ null mutant we performed interaction experiments in the presence of the *N*-linked mannan core Man_9_GlcNAc_2_ (M9) (Mora-Montes et al., 2004) and measured the cytokine production. Human PBMCs pre-incubated with M9 and then challenged with live WT cells stimulated similar cytokine levels than cells without the preincubation step; however, in similar experiments using HK cells the amount of TNFα, IL-6 and IL-1β was significantly diminished, when compared with the system without M9 (Figure 7 and data not shown). For the case of IL-10, pre-incubation with M9 did not affect the cytokine levels stimulated by either live or HK cells (Figure 7). In the case of both live and HK *och1*Δ null mutant cells, the levels of TNFα, IL-6, and IL-1β were significantly reduced upon pre-incubation with M9 (Figure 7 and data not shown). As the WT cells, the levels of IL-10 stimulated with either live and HK *och1*Δ null mutant cells were not affected by pre-incubation with M9 (Figure 7). The reintegrant control strain stimulated similar cytokine levels to those observed with the WT strain (Figure 7). The *pmr1*Δ null mutant showed a similar ability to stimulate cytokine production than the *och1*Δ null strain (data not shown). This antagonistic ability of M9 during cytokine production suggested the involvement of mannose receptor (MR) in the PBMC-yeast interaction, as this pattern recognition receptor senses branched oligosaccharides with alpha glycosidic bonds, as M9 (Netea et al., 2006). Therefore, we explored the contribution of this receptor in the *C. tropicalis* recognition. Human PBMCs preincubated with anti-MR antibodies were challenged with either WT or mutant cells. The anti-MR antibodies did not affect the ability to produce IL-10 but negatively affected the TNFα, IL-6, and IL-1β levels stimulated with HK WT cells (Figure 7 and data not shown). For the case of the *och1*Δ null mutant, the presence of anti-MR antibodies within the system had little impact on the production of IL-10 but reduced the production of TNFα, IL-6, and IL-1β stimulated with live cells (Figure 7 and data not shown). Although the pro-inflammatory cytokine levels were reduced in the presence of anti-MR and HK cells of the *och1*Δ null mutant, this was not statistically significant (Figure 7 and data not shown). The control system with an isotype-matched antibody or with the reintegrant control strain shown cytokine levels similar to those stimulated with the WT cells.

Finally, we assessed the contribution of dectin-1 during the interaction with *och1*Δ null cells. As in the case of the WT strain, the TNFα, IL-6, IL-1β, and IL-10 levels stimulated by either live or HK mutant cells was significantly reduced (Figure 7 and data not shown). Similarly, the cytokine production stimulated by live or HK *pmr1*Δ cells was negatively affected by pre-incubation with laminarin (data not shown), stressing the importance of dectin-1 engagement with β1,3-glucan during the *C. tropicalis*-human PBMC interaction.

### *PMR1* and *OCH1* Are Required for *Candida tropicalis* Virulence in Both *Galleria mellonella* and the Mouse Model of Systemic Candidiasis

**FIGURE 8:**
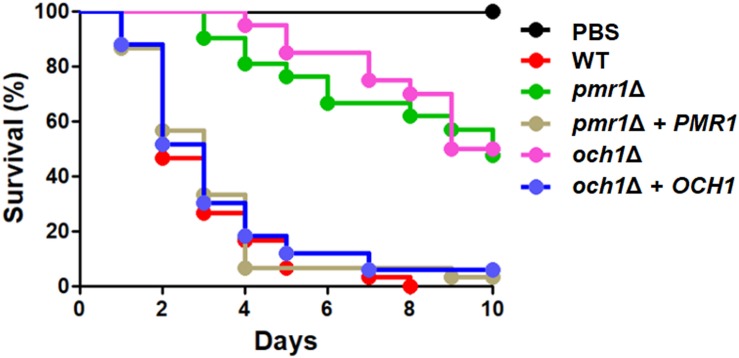
Mortality curve of *Galleria mellonella* larvae infected with either the *Candida tropicalis pmr1*Δ or *och1*Δ null mutant strains. Each larva was infected with 2 × 10^7^ yeast cells and survival was monitored daily. Experiments were performed three times, with a total of 30 larvae per group (10 larvae for each experiment). PBS, control group injected only with PBS. Strains used are MYA-3404 (WT), HMY207 (*pmr1*Δ), HMY208 (*pmr1*Δ + *PMR1*), HMY181 (*och1*Δ), and HMY205 (*och1*Δ + *OCH1*). The null mutant strains showed a significant difference in the ability to kill larvae when compared to the parental or reintegrant control strains (*P* < 0.05 in all cases).

To assess the relevance of protein mannosylation in the *C. tropicalis* virulence, we first infected larvae of *G. mellonella* and evaluated the survival rate of these animals. The animals infected with WT and the reintegrant control strains showed similar survival curves, with the total of the animal population dead after 8 days of infection when injected with the WT strain (Figure 8). For the case of the reintegrant strains, the total of the animal population succumbed after 10 days post-infection, and this difference was not statistically significant (Figure 8; *P* = 0.19 and 0.27, when the curve associated to WT cells is compared with either *pmr1*Δ + *PMR1* or *och1*Δ + *OCH1*, respectively). The survival curves of animals infected with either the *pmr1*Δ or the *och1*Δ null mutant were similar and showed virulence attenuation, with 50% of the animal population surviving at the end of the observation period (Figure 8). The fungal burdens in the inoculated animals indicated a defect in the null mutant strains to colonize the host tissues, as this was lower than that associated to the WT or the reintegrant control strains (1.3 × 10^7^ ± 0.6 × 10^7^ cells mL^–1^, 0.7 × 10^7^ ± 0.2 × 10^7^, 1.1 × 10^7^ ± 0.4 × 10^7^cells mL^–1^, 0.6 × 10^7^ ± 0.4 × 10^7^, 1.0 × 10^7^ ± 0.6 × 10^7^cells mL^–1^ for WT, *pmr1*Δ, *pmr1*Δ + *PMR1*, *och1*Δ, *och1*Δ + *OCH1*, respectively. *P* < 0.05 when compared to the null mutant strains with the WT control strain).

**FIGURE 9:**
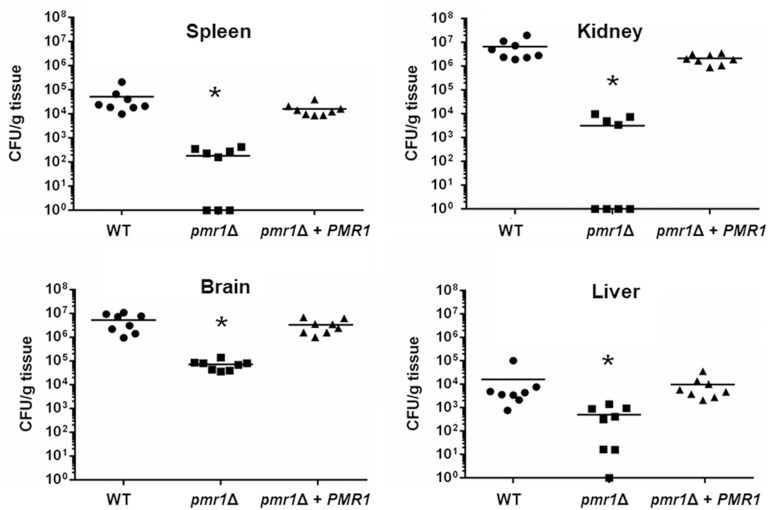
The *Candida tropicalis pmr1*Δ null mutant has decreased virulence in a mouse model of non-lethal systemic candidiasis. Wild-type Balb/c mice were infected with 1 × 10^6^ yeast cells and the fungal burdens in the spleen, kidneys, brain, and liver were determined after 3 days of infection. Data are expressed as colony forming units (CFU) g^–1^ tissue (mean). Results are pooled data from two separate experiments with eight mice per group. ^∗^*P* < 0.05 when compared to animals infected with the WT control strain. Strains used are MYA-3404 (WT, closed circle), HMY207 (*pmr1*Δ, closed square), and HMY208 (*pmr1*Δ + *PMR1*, closed triangle).

**FIGURE 10:**
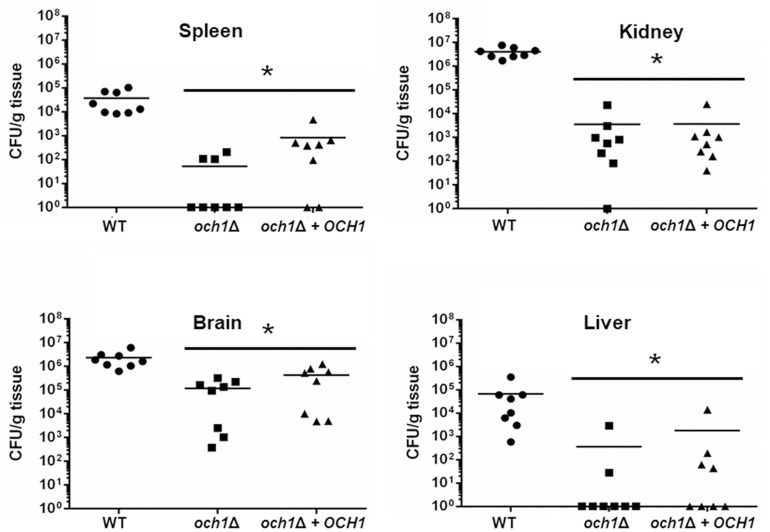
The *Candida tropicalis och1*Δ null mutant has decreased virulence in a mouse model of non-lethal systemic candidiasis. Same legend as Figure 9, but strains used are MYA-3404 (WT), HMY207 (*pmr1*Δ), HMY208 (*pmr1*Δ + *PMR1*). MYA-3404 (WT, closed circle), HMY181 (*och1*Δ, closed square), and HMY205 (*och1*Δ + *OCH1*, closed triangle). ^∗^*P* < 0.05, when compared to animals infected with the WT control strain.

We next used a murine model of non-lethal disseminated candidiasis as previously reported (Ifrim et al., 2014; Navarro-Arias et al., 2016; Perez-Garcia et al., 2016), to evaluate the virulence in this *in vivo* setting. After 3 days of infection, BALB/c mice infected with the *pmr1*Δ null mutant had lower fungal burdens in the spleen, kidneys, brain, and liver, when compared to either the WT control cells or the reintegrant control strain (Figure 9). Similarly, the fungal burden of animals infected with the *och1*Δ null mutant strain was lower to that observed in animals infected with the WT strain, but interestingly, the *och1*Δ + *OCH1* reintegrant control strain behaved like the null mutant strain, and fewer colony-forming units were recovered from the spleen, kidneys, brain, and liver of infected animals (Figure 10). Collectively, these results indicate that *PMR1* and *OCH1* have a significant role in the interaction of *C. tropicalis* with either *G. mellonella* or the mouse.

## Discussion

So far, the knowledge we have about the genetic machinery behind protein glycosylation and cell wall assembly in *C. tropicalis* is limited, likely because of the close genetic relationship with *C. albicans*, a thoroughly studied model of fungal cell wall synthesis. Previously, we have genetically addressed the relevance of protein phosphomannosylation during the *C. tropicalis*-host interaction and found that the role of this wall component in the interaction with macrophages is different from that described in *C. albicans* (Hernandez-Chavez et al., 2018). This report represents the first genetic approach to address the relevance of *C. tropicalis N*- and *O*-linked mannan for cell wall composition, organization, and interaction with the host.

The *O*-linked mannosylation pathway includes the Pmt and Mnt transferases that belong to gene families composed each of team of five members with functional redundancy (Mora-Montes et al., 2009; Martinez-Duncker et al., 2014). This has represented a challenge to assess the contribution of each of the family members to both *O*-linked mannan elaboration and the study of the host-fungus interaction (Munro et al., 2005; Prill et al., 2005; Mora-Montes et al., 2010). Therefore, we chose to disrupt *PMR1* to get indirect insights on the contribution of *O*-linked mannans to the *C. tropicalis*-host interaction.

The bioinformatics analysis and the heterologous complementation in *C. albicans* strongly suggest that the ORFs we have analyzed are indeed the functional orthologs of *C. albicans PMR1* and *OCH1.* The *C. albicans* genome also contains *HOC1*, a gene encoding for a mannosyltransferase that is part of the M-Pol II complex, involved in the elongation of the α1,6-backbone of the *N*-linked mannan outer chain (Martinez-Duncker et al., 2014). Both *OCH1* and *HOC1* encode products with significant similarity in the primary sequences but they are unable to complement each other and have signature sequences that differentiate them from each other (Lambou et al., 2010; Lozoya-Perez et al., 2019; see Supplementary Figure S4). Bioinformatics analysis of the *C. tropicalis* genome could identify the putative gene encoding Hoc1, whose primary structure is closer to *C. albicans Hoc1* than *Och1*. Moreover, a sequence analysis differentiated Och1 proteins from Hoc1 proteins, being grouped in different clades (Supplementary Figure S4). Therefore, it is unlikely that we have identified the functional ortholog of *HOC1* instead of *OCH1*. In addition, if this *C. tropicalis* ORF was the true ortholog of *HOC1*, it would be difficult to explain the phenotype restoration of the *C. albicans och1*Δ null mutant expressing *C. tropicalis HOC1*, as the M-Pol II complex works after Och1 has added the first mannose residue to the *N*-linked mannan outer chain backbone (Martinez-Duncker et al., 2014).

Both the *C. tropicalis pmr1*Δ and *och1*Δ null mutants displayed phenotypical traits characteristic of yeast mutants with defects in the protein mannosylation pathways: increased doubling rates, abnormal cell and colony morphology, decreased mannan content, changes in the cell wall composition and organization, and fitness to resist the action of wall perturbing agents (Bates et al., 2005, 2006, 2013; Munro et al., 2005; Mora-Montes et al., 2007, 2010; Hall et al., 2013; Navarro-Arias et al., 2016; Perez-Garcia et al., 2016). In *Candida guilliermondii*, loss of *PMR1* affected the cellular growth, changing from a radial growth that maintains the yeast cell shape, to a unilateral one, establishing the growth of pseudohyphae (Navarro-Arias et al., 2016). Our results are in line with those found in *C. albicans* and *Saccharomyces cerevisiae*, where cells grow as yeast cells upon *PMR1* disruption (Antebi and Fink, 1992; Bates et al., 2005), stressing that *C. guilliermondii* has different mechanisms to mobilize calcium within the cell to those found in *C. albicans* or *C. tropicalis*.

Interestingly, the changes in the composition and organization of structural polysaccharides were not similar in the mutant strains under study, but comparable to those reported in *C. albicans* (Bates et al., 2005, 2006). One discrepancy between *C. albicans* and *C. tropicalis* though is the chitin content in the *och1*Δ null mutant strains, as its abundance in the former species was doubled but not affect in the *C. tropicalis* strain (Bates et al., 2006). Since all the components of the core of the cell wall integrity pathway (the master regulator to control the wall composition and for triggering compensatory mechanisms when this structure is affected) are present within the *C. tropicalis* genome (Butler et al., 2009), it is likely this controls the cell wall remodeling upon aggression by stressors or genetic modifications that affect the cell wall structure (Dichtl et al., 2016). It has been described that the repertoire of receptors that activate this signaling pathway and its ability to engage with ligands is species-specific (Dichtl et al., 2016), which may offer an explanation to the differential effect of *OCH1* loss on the chitin content in both *C. albicans* and *C. tropicalis*. In both cases, the decreased amount of mannan content may account for the exposure of this wall polymer on the surface.

It was anticipated that the *och1*Δ null mutant had defects only in the *N*-linked mannan synthesis; while the *pmr1*Δ mutant in both *N*- and *O*-linked mannan synthesis, a fact that was confirmed in our study. Therefore, it is feasible to hypothesize that *O*-linked mannans have a significant role in the signaling pathways that control the cell wall integrity pathway (Levin, 2005). Despite the contribution of *N*-linked mannans in the activation of this pathway has been largely documented, the detailed mechanisms behind the contribution of *O*-linked mannans to this signaling pathway are poorly understood (Levin, 2005).

As reported in *C. albicans* (Netea et al., 2006; Gow et al., 2007), *C. tropicalis O*-linked mannans were dispensable for stimulation of cytokine production, being *N*-linked mannans and β1,3-glucan the major stimuli. In the case of IL-10 stimulation, we found that mannans were not required for this cytokine production, in fact, removal of these cell wall components had a positive effect on the IL-10 levels. It has been reported that different to other cytokines, engagement of dectin-1 with β1,3-glucan is enough to signaling the production of IL-10, representing a mannan-independent pathway for stimulation of this anti-inflammatory cytokine (Reid et al., 2009). Our results confirm this mannan-independent mechanism of IL-10 production also occurs in the *C. tropicalis*-PBMC interaction. The activation of this signaling pathway is the most likely explanation to the fact that the *och1*Δ and the *pmr1*Δ null mutants were capable of inducing more IL-10 production that the control strains in the live form, as most of the β1,3-glucan was exposed on the cell surface of the mutants. This increment in the amount of superficial β1,3-glucan is also the likely explanation of the increased ability of the live mutant strains to stimulate higher levels of proinflammatory cytokines, stressing once again that the major fungal player in cytokine production is β1,3-glucan. Alternatively, it is possible to speculate that removal of mannans by disruption of either *OCH1* or *PMR1* increments the exposure of cell wall phospholipomannan on the cell surface, accounting for the increased ability to stimulate cytokine production. The presence of phospholipomannan has been described in the cell wall of both *C. albicans* and *C. tropicalis* (Cantelli et al., 1995). In the former, it has been demonstrated that stimulates pro-inflammatory cytokines via engagement with TLR2 and galectin-3 (Jouault et al., 2003, 2006). Further experiments are required to assess the contribution of phospholipomannan in the ability of the *och1*Δ and *pmr1*Δ mutants to stimulate cytokine production. Nonetheless, our results clearly suggest that like in other *Candida* species *C. tropicalis* mannans are masking β1,3-glucan, precluding interaction with dectin-1 (Wheeler and Fink, 2006; Gow et al., 2007; Navarro-Arias et al., 2016; Perez-Garcia et al., 2016). It is worthy of note that despite the null mutant cells have more β1,3-glucan exposed at the cell surface, this did not lead to a higher stimulation of pro-inflammatory cytokines, even after heat inactivation of cells. Similar observations have been reported for *C. albicans*, *C. parapsilosis*, and *C. guilliermondii* null mutants with defects in mannan elaboration and higher levels of β1,3-glucan at the cell wall (Netea et al., 2006; Mora-Montes et al., 2007, 2010; Navarro-Arias et al., 2016; Perez-Garcia et al., 2016). Our results showed that MR plays a role as important as that described for dectin-1 in the stimulation of the proinflammatory cytokines analyzed in this work, and our observations point out that this receptor engages with *N*-linked mannans, as reported in *C. albicans* (Netea et al., 2006). Therefore, it is tempting to speculate that even though signaling via dectin-1 is important for cytokine production, the interaction of this lectin with its ligand should be part of a co-stimulation network where other receptors, or at least MR, are involved in cytokine production. In line with this hypothesis, it has been reported the synergistic interaction between dectin-1 with TLR-2, TLR-4, TLR-5, TLR-7 or TLR-9 during cytokine stimulation (Reid et al., 2009).

It is worthy of mention that despite the fact that β-elimination can remove *O*-linked mannans from the wall, this alkali treatment could affect protein structures and can be released to the medium (Mormeneo et al., 1994), potentially affecting the outcome of the immune cell-fungus interaction. However, no pattern-recognition receptor on the surface of immune cells has been described to engage with the polypeptide backbone of a protein cell wall (Netea et al., 2008).

Another interesting observation is the fact that the *C. albicans och1*Δ null mutant barely stimulates cytokine production (Bates et al., 2006), whereas here, loss of *OCH1* had a mild effect on the ability of *C. tropicalis* to stimulate cytokine production, highlighting a subtle but different relevance of *N*-linked mannans in cytokine stimulation by these species. We found that the presence of the *N*-linked mannan core on the *C. tropicalis* surface is likely to account for this observation and that this oligosaccharide has the ability to block the stimulation of cytokines most likely via MR. It was reported that in *C. albicans* the *N*-linked mannan core is further modified in the *och1*Δ null mutant with two to seven α1,2-mannose units (Bates et al., 2006), which contrast with the glycans isolated from *S. cerevisiae och1*Δ null mutant that added only one mannose unit to the *N*-linked mannan core (Bates et al., 2006). It is tempting to speculate that in *C. tropicalis* this *N*-linked mannan core is elongated with more mannose residues than in *C. albicans*, which accounts for its ability to stimulate higher cytokine levels to that observed with *C. albicans*. If this was true, the number of mannose units modifying the *N*-linked mannan core and the kind of glycosidic linkages involved remain to be addressed.

Both null mutant strains showed decreased virulence in both the *G. mellonella* and murine models of systemic candidiasis, similar to other *Candida* species where mutants in either *OCH1* or *PMR1* have been generated (Bates et al., 2005; Navarro-Arias et al., 2016; Perez-Garcia et al., 2016). However, in the mouse model, the reintegrant control strain for *OCH1* failed to colonize as the WT control strain. Since in all the phenotypical analysis this strain behaved like the WT control strain, it is unlikely this could be related to the generation of the strains. Haploinsufficiency has been described in *C. albicans* (Chaillot et al., 2017), and a significant reduction of mannosyltransferase activity has been reported for the heterozygous strain in *OCH1* (Bates et al., 2006). Therefore, we hypothesize that in *C. tropicalis* the gene could be haploinsufficient and the enzyme activity provided by one *OCH1* copy could be enough to display a normal phenotype under the conditions tested but insufficient to adapt to the mouse milieu. Nevertheless, our results clearly showed virulence attenuation in the mouse model upon *OCH1* disruption. Since both mutant strains showed defects in the doubling time and wall fitness, it is likely the whole-cell fitness is compromised by disruption of any of the genes under study, which is likely to account to the defects of the null mutants to interact with either *G. mellonella* or the mice, in other words, the virulence defect is most likely due to inability to properly adapt to the *in vivo* milieu than the loss of virulence factors.

In conclusion, we report the identification and disruption of *C. tropicalis PMR1* and *OCH1*. These genes contribute to protein mannosylation in *C. tropicalis* and are relevant for fungal virulence, cell wall composition, assembly, and fitness. In addition, *N*- and *O*-linked mannans have differential roles for cytokine stimulation during the *C. tropicalis*-human PBMC interaction.

## Materials and Methods

### Strains and Growth Conditions

Organisms used in this study are listed in Table 2. Unless otherwise indicated, cells were maintained and propagated at 28°C in YPD medium [2% (w/v) bacteriological peptone, 1% (w/v) yeast extract, 2% (w/v) glucose]. The medium was supplemented with 2% (w/v) agar when the solid plates were required. During gene disruption, the *SAT1* marker was recycled by growing cells at 28°C in liquid YEP [2% (w/v) bacteriological peptone and 1% (w/v) yeast extract]. To prepare cells for interaction with the host and cell-wall analysis, the strains were grown at 30°C in 500 mL flasks containing 100 mL of fresh medium with shaking at 200 rpm until reaching the logarithmic growth phase. Cells were inactivated by incubating at 56°C for 60 min. The confirmation of the presence of non-viable cells was performed on YPD plates incubated for 4 days at 28°C. For chemical remotion of *O*-linked mannans, cells were β-eliminated in 10 mL of NaOH 0.1 N and incubated at room temperature during 18 h, as previously described (Diaz-Jimenez et al., 2012). Cell suspensions were neutralized with HCl 0.1 N, and pelleted at 2000 × g, for 5 min. The supernatant was saved for *O*-linked mannans analysis (see below). The enzymatic *N*-linked mannan removal was performed with 25 U endo H (New England Biolabs) as reported (Mora-Montes et al., 2012). In both cases, cells were washed twice with sterile phosphate-buffered saline (PBS) and viability was confirmed by quantifying the colony-forming units before and after the treatment. The loss of viability after β-elimination and endo H treatment was 1.8 ± 0.9% and 3.3 ± 4.1%, respectively). To disperse cell aggregates, 2 units mL^–1^ chitinase (Sigma) was added to medium when required (Bates et al., 2006; Perez-Garcia et al., 2016).

### Heterologous Complementation in *Candida albicans*

The *PMR1* ORF was amplified by PCR using the primer pair 5′-AAGCTTATGAGTGATAACCCTTATGAACTA-3′ and 5′-GCTAGCTTATACTCCATATGTATAATTATTAGTATAAATAG T-3′ (underlined sequences correspond to restriction sites for *Hin*dIII and *Nhe*I, respectively); while *OCH1* ORF was amplified with the primer pair 5′-AAGCTTATG CGTCTGAAGGATATCA-3′ and 5′- GCTAGCTTAATCTTCC ATTTCTGGCAT (underlined sequences correspond to restriction sites for *Hin*dIII y *Nhe*I, respectively). The amplicons were cloned into pCR^®^2.1-TOPO^®^ (Invitrogen), and subcloned into the *Hin*dIII and *Nhe*I sites of pACT1 (Barelle et al., 2004), generating pACT1-Ct*PMR1* and pACT1-Ct*OCH1*. A *C. albicans pmr1*Δ null mutant (Bates et al., 2005) was transformed with *Stu*I-digested pACT1-Ct*PMR1*, generating strain HMY185; whereas a *C. albicans och1*Δ null mutant (Bates et al., 2006) was transformed with *Stu*I-digested pACT1-Ct*OCH1*, generating the strain HMY148. Since the *OCH1* ORF contains an internal recognition site for *Hin*dIII, after cloning into pCR^®^2.1-TOPO^®^ (Invitrogen), the codon ^450^AAA^453^ was modified to ^450^AAG^453^ by site-directed mutagenesis using the Phusion Site-Directed Mutagenesis Kit (Thermo) and the primer pair 5′- CCTGATGTTTCAAGGCTTATAAAATTATGCCAAAATC-3′ and 5′- GGCATAATTTTATAAGCTTTGAAACATCAGGAA TTTC-3′. The mutation was confirmed by DNA sequencing, before cloning into pACT1.

### Construction of Null Mutant and Reintegrant Control Strains

*PMR1* and *OCH1* were deleted using the *SAT1* flipper method, as previously described (Hernandez-Chavez et al., 2018). For both genes, the 1500 bp upstream and the 1500 bp downstream regions of the ORF were amplified by PCR and cloned into the *Apa*I-*Xho*I and *Not*I-*Sac*I sites of pSFS2 (Reuß et al., 2004), respectively, generating the corresponding disruption cassettes. The primer pairs used for the generation of the disruption cassettes were: for the *PMR1* upstream region 5′-GGGCCCAGTTGATTGAAAAATTTTGGCCAGG-3′ and 5′- CTCGAGAGGGAGTCTGTTGAAGGAGTG; for the *PMR1* downstream region 5′-GCGGCCGCACTTCCAGAAG TGTGGTATGTGT-3′ and 5′- GAGCTCACTACGCATACCT TCCAACCA-3′; for the *OCH1* upstream region 5′-GGGCCCACACAACCGTCTCTATCAGG-3′ and 5′-CTCGAG TGTTAACTTCTGAATTGGTCTATGA-3′; and for the *OCH1* downstream region 5′- GCGGCCGCTCTAATGTTCACAA TTAAAACTTGTATT-3′ and GAGCTCGATTTGTTAATTAA AGAAGATGGTGT-3′. The disruption cassettes were excised from the vector by digesting with *Apa*I and *Sac*I and used for cell transformation of strain ATCC^®^ MYA-3404 (referred to as WT control strain). The selection of transformed cells was performed in YPD plates added with 200 μg mL^–1^ nourseothricin (Goldbio). The integration of the disruption cassettes in the targeted locus was analyzed by PCR with primer pairs aligning inside and outside of the recombinations regions. Cells were grown for 2 days at 28°C in YEP broth added with 2% (w/v) maltose to recycle the *SAT1* marker. The loss of *SAT1* was confirmed by plating cells in YPD supplemented with 10 μg mL^–1^ nourseothricin and PCR. The second allele of *PMR1* and *OCH1* was disrupted in the second round of transformation with the same cassettes and recycling of the *SAT1* marker.

To reintegrate *PMR1* or *OCH1* into the *C. tropicalis* null mutant generated, the ORF plus 1000 bp upstream and 500 bp downstream was amplified by PCR using the following primer pairs: 5′- GGGCCCCCAATGTTATTACGTCCTGGTGG-3′ and 5′- CTCGAGGAGGGAGAAAAGGGGAGGATAC-3′ for *PMR1*, and 5′- GGGCCCGGTAATGCTTCACCATCATCAACT-3′ and 5′- CTCGAGATCCACGGAAAGAACCGCAA-3′ for *OCH1*. Either the 2789 bp fragment corresponding to *OCH1* or *PMR1*, a 4517 bp amplicon, were cloned into the *Apa*I-*Xho*I sites of pSFS2, and then a 700 bp fragment downstream of the disrupted locus was cloned into the *Not*I-*Sac*I sites of the same vector. These downstream fragments were generated by PCR using the primer pairs 5′-GCGGCCGCATTGCTTCATTTGGAGGTGTT G-3′ and 5′-GAGCTCCTACGCATACCTTCCAACCA-3′ for *PMR1*; and 5′-GCGGCCGCCCTGGAGGCAAGTGCTTTTCA-3′ and 5′-GAGCTCACGGATTTGTATTTGTCAAGAGCCA-3′ for *OCH1*. These reintegration constructions were linearized with *Apa*I and *Sac*I and used to transform the null mutant strains. Upon recycling of the *SAT1* marker, confirmation of the reintegration of either *PMR1* or *OCH1* in one of the native loci was confirmed by PCR.

### Cell Wall Analysis

For the analysis of the composition, grown-cells in YPD were disrupted in a Precellys homogenizer (Bertin), the homogenate was centrifuged, the pellet saved, thoroughly washed with deionized water, and the cell walls were cleansed and acid-hydrolyzed as described previously (Mora-Montes et al., 2007). The acid-hydrolyzed samples were analyzed by HPAEC-PAD in a carbohydrate analyzer system (Thermo), using the columns and separation conditions previously reported (Plaine et al., 2008). The cell wall protein content was measured upon alkali hydrolysis of the cell wall and the Bradford method, as previously described (Mora-Montes et al., 2007).

The cell wall porosity was determined by the relative porosity of polycations, as reported earlier (De Nobel et al., 1990). Cells were grown in YPD broth at 28°C until reaching the mid-log phase, washed twice with PBS, and aliquots containing 1 × 10^8^ cells were suspended in either 10 mM Tris–HCl, pH 7.4 (buffer A), buffer A plus 30 μg/mL poly-L-lysine (Mw 30–70 kDa, Sigma) or buffer A plus 30 μg/mL DEAE-dextran (MW 500 kDa, Sigma), and incubated for 30 min at 30°C with constant shaking at 200 rpm. Cell suspensions were pelleted by centrifuging, the supernatants saved, further centrifuged, and used to measure the absorbance at 260 nm. The cell wall porosity was calculated as described previously, using the porosity to poly-L-lysine for data normalization (De Nobel et al., 1990). To quantify the cell wall phosphomannan, the cellular ability to bind Alcian blue was analyzed as described (Hobson et al., 2004).

Localization of mannan, chitin, and β1,3-glucan within the cell wall was analyzed by fluorescent microscopy using 1 mg mL^–1^ Con A-FITC (Sigma), 1 μg mL^–1^ WGA-FITC (Sigma) and 5 μg/mL IgG Fc-Dectin-1 chimera conjugated with anti-Fc IgG-FITC for mannan, chitin, and β1,3-glucan staining, respectively (Graham et al., 2006; Mora-Montes et al., 2011; Marakalala et al., 2013). Cells were examined by fluorescence microscopy using a Zeiss Axioscope-40 microscope and an Axiocam MRc camera. The fluorescence was quantified from the pictures acquired with Adobe Photoshop^TM^ CS6 using the formula: [(total of green pixels-background green pixels) × 100]/total pixels. The experiment was performed three independent times, with a total of 300 cells analyzed per strain.

### Quantification of Cell Wall *N*-Linked and *O*-Linked Mannans

Cells grown at the mid-log phase in YPD were incubated overnight at 37°C with 25 U endo H (New England Biolabs) to trim cell wall *N-*linked mannans (Mora-Montes et al., 2012). Cells were also β-eliminated with 0.1 N NaOH, as described (Diaz-Jimenez et al., 2012). Then, cells were pelleted, and the supernatant used to determine the mannose content with the phenol-sulfuric-acid method (Dubois et al., 1956). In both cases, mannan release was confirmed by HPAEC-PAD as reported (Perez-Garcia et al., 2016).

### Analysis of Susceptibility to Cell Wall Perturbing Agents

Cell growth in the presence of cell wall perturbing agents was performed as previously described (Navarro-Arias et al., 2016). Briefly, overnight grown cells in YPD were washed with deionized water, separated using a syringe with a 32-gauge needle and suspended at an OD_600__nm_ = 1.0. Then, fresh YPD broth was inoculated with the fungal cells at an OD_600__nm_ = 0.01, and aliquots containing 95 μL were placed into 96-well plates. The cell wall perturbing agents, in a final volume of 5 μL, were added to each well. Mock wells contained 5 μL of the vehicle only and were used to normalize the results. The final OD_600__nm_ for each well was quantified after 24 h incubation at 30°C. Calcofluor white and Congo red were from Sigma; whereas hygromycin B from GoldBio. Growth data were normalized as a percentage of those generated with the mock interactions.

### Ethics Statement

The use of human PMBCs was approved by the Ethics Committee from Universidad de Guanajuato (permission number 17082011), and peripheral blood samples were withdrawn from healthy adult volunteers after information of the study was disclosed and a written consent form signed. The Ethics Committee at the University of Szeged approved the experimentation with mice (permission number XVI./3646/2016).

### Cytokine Production by Human PBMCs

Upon peripheral blood was collected in tubes containing EDTA as anticlotting, PBMCs were isolated by differential centrifugation using Histopaque-1077 (Sigma), as reported (Endres et al., 1988). To stimulate cytokine production, 100 μL containing 5 × 10^5^ PBMCs in RPMI 1640 Dutch modification (added with 2 mM glutamine, 0.1 mM pyruvate and 0.05 mg mL^–1^ gentamycin; all reagents from Sigma) were plated onto round-bottom 96-well microplates, and then 100 μL containing 1 × 10^5^ fungal cells freshly harvested or treated were added to each well. The plates were incubated for 24 h at 37°C with 5% (v/v) CO_2_. In some experiments, PBMCs were pre-incubated for 1 h at 37°C with 200 μg mL^–1^ laminarin (Sigma), 10 μg mL^–1^ M9 (Man_9_GlcNAc_2_; Sigma), 10 μg mL^–1^ anti-MR (Invitrogen, Cat. No. Mab-Hmr), or 10 μg mL^–1^ IgG1 antibodies (Santa Cruz Biotechnology) before interaction with fungal cells. All these reagents were LPS free, as tested with the *Limulus* amebocyte lysate (Sigma, data not shown). Nonetheless, interactions were conducted in the presence of 5 μg mL^–1^ polymyxin B (Sigma) (Navarro-Arias et al., 2016). The plates were centrifuged for 10 min at 3000 × *g* at 4°C, and the supernatants were collected and stored at −20°C until used.

The concentrations of TNFα, IL-6 and IL-10 were quantified with the ABTS ELISA Development kits from Preprotech; while IL-1β levels were measured with a DuoSet ELISA Development kit (R&D Systems).

### Infection of *G. mellonella* Larvae

The *G. mellonella* infection was performed as previously reported (Perez-Garcia et al., 2016). Briefly, 2 × 10^7^ yeast cells contained in 10 μL PBS were passaged through a syringe with a 32-gauge needle, the last left pro-leg of the larva sanitized and fungal cells directly injected into the hemolymph, using a Hamilton syringe and a 26-gauge needle. Upon infection, the animals were kept at 37°C and survival was monitored daily. Animal death was defined by extense melanization and irritability absence. Each experimental group contained 30 larvae. Mock infections with animals injected with PBS were included as a control. The content of colony-forming units was determined by incubating serial dilutions of the hemolymph on YPD plates for 28°C for 72 h.

### Infection of Mice and Analysis of the Fungal Burden

A non-lethal experimental model of disseminated candidiasis was performed as reported (Ifrim et al., 2014; Perez-Garcia et al., 2016). Animal groups containing 8 female Balb/c WT mice (older than 12 weeks old and 20 to 25 g of weight) were injected via the lateral tail vein with 100 μL of sterile PBS containing 1 × 10^6^ *C. tropicalis* yeast cells, previously passaged through a syringe with a 29-gauge needle. As a control group, eight mice were injected with 100 μL of sterile PBS. Animals having access to sterile water and a normal diet *ad libitum* were monitored daily and showed no signs of infection such as weight loss, lethargy, ruffled fur or rapid shallow breathing during 3 days following the intravenous injection. At this point, animals were euthanatized, and the liver, brain, kidneys, and spleen were removed, weighed, and separately homogenized with a tissue grinder. The fungal burden was determined by incubating serial dilutions on YPD agar plates for 28°C for 48 h.

### Statistical Analysis

Statistical analysis was performed using GraphPad Prism 6 software. Growth data in the presence of cell wall perturbing agents were analyzed by two-way ANOVA. Cytokine stimulation using human PMBCs was performed in duplicate with seven healthy donors, whereas the rest of the *in vitro* experiments were performed at least thrice in duplicate. Data represent the cumulative results of all experiments performed. The Mann–Whitney *U* test or unpaired *t*-test was used to establish statistical significance (see figure legends for details), with a significance level set at *P* < 0.05. Experiments with *G. mellonella* were performed three times, with a total of 30 larvae per group (10 larvae for each experiment). Results were analyzed using the Log–rank test and arranged in survival curves using Kaplan-Meier charts. The statistical significance was set at *P* < 0.05.

## Data Availability Statement

The datasets generated for this study are available on request to the corresponding author.

## Ethics Statement

The studies involving human participants were reviewed and approved by the Ethics Committee from Universidad de Guanajuato. The patients/participants provided their written informed consent to participate in this study. The animal study was reviewed and approved by the Ethics Committee at the University of Szeged.

## Author Contributions

MH-C, AG, IM-D, and HM-M designed and conceived the study. MH-C, DC-G, ÁN, NL-P, JM-Á, RS-M, and NH performed the experiments. MH-C, DC-G, NL-P, JM-Á, RS-M, NH, AG, IM-D, and HM-M analyzed the data. AG and HM-M wrote the manuscript. All authors approved the final version of the manuscript.

## Conflict of Interest

The authors declare that the research was conducted in the absence of any commercial or financial relationships that could be construed as a potential conflict of interest.

## Footnotes

1 http://www.ncbi.nlm.nih.gov/
