# Supplementary material for: Role of Protein Mannosylation in the Candida tropicalis-Host Interaction
Source: Front Microbiol. 2019 Nov 28;10:2743. doi: 10.3389/fmicb.2019.02743 (PMC6892782; doi:10.3389/fmicb.2019.02743)
Supplement: Supplementary file 1 [file Data_Sheet_1.docx]

Supplementary Material

**Role of Protein Mannosylation In the *Candida tropicalis*-Host Interaction**

**Marco J. Hernández-Chávez^1^, Diana M. Clavijo-Giraldo^1^, Ádám Novák^2^, Nancy E. Lozoya-Pérez^1^, José A. Martínez-Álvarez^1^, Roberta Salinas-Marín^3^, Nahúm V. Hernández^1^, Iván Martínez-Duncker^3^, Attila Gácser^2,4,^*, and Héctor M. Mora-Montes^1,^***

^1^Departamento de Biología, División de Ciencias Naturales y Exactas, Campus Guanajuato, Universidad de Guanajuato, Noria Alta s/n, col. Noria Alta, C.P. 36050, Guanajuato, Gto., México

^2^Department of Microbiology, University of Szeged, Közép fasor 52, H-6726 Szeged, Hungary ^3^Laboratorio de Glicobiología Humana y Diagnóstico Molecular; Universidad Autónoma del Estado de Morelos, Cuernavaca, Morelos, México.

^4^MTA-SZTE "Lendület" Mycobiome” Research Group, University of Szeged, Szeged, Hungary

*** Correspondence:**Corresponding Authors: **Attila Gácser** [**gacsera@bio.u-szeged.hu**](mailto:gacsera@bio.u-szeged.hu)**; Héctor M. Mora-Montes** [**hmora@ugto.mx**](mailto:hmora@ugto.mx)

# Supplementary Data


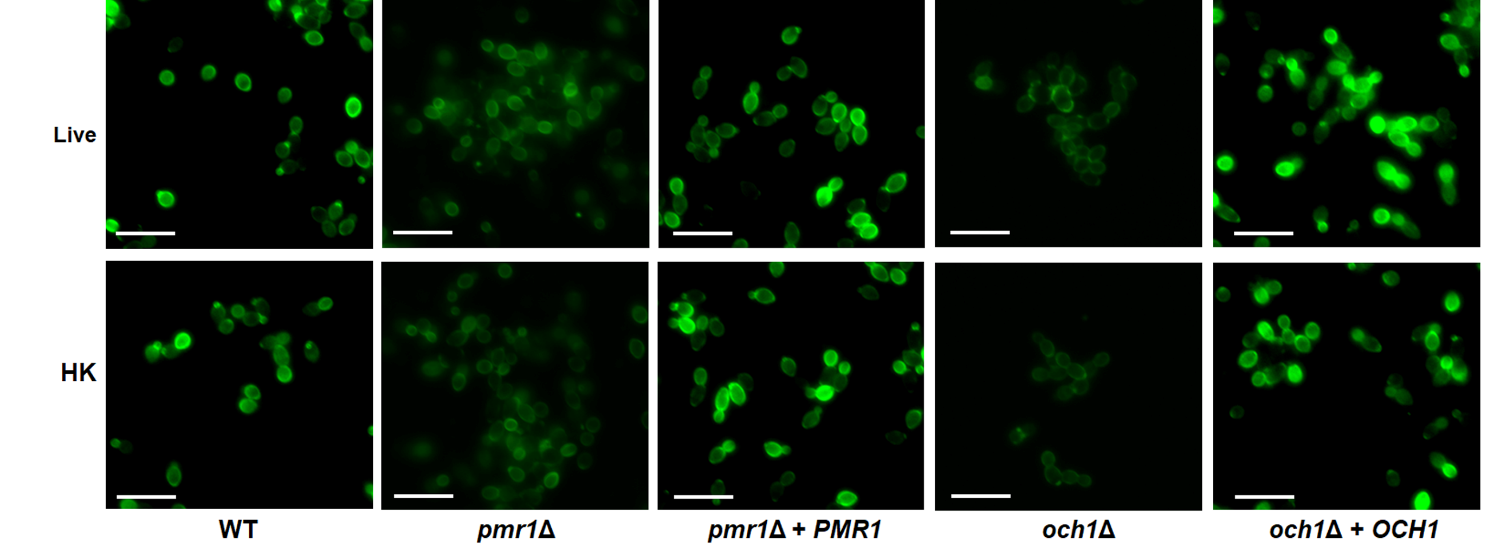


**Figure S1: The labeling of cell wall mannan is reduced in the *C. tropicalis* *pmr1*Δ and *och1*Δ null mutants.** Live or heat-killed (HK) cells were incubated with fluorescein isothiocyanate-concanavalin A conjugate as described in the Experimental procedures and inspected under fluorescence microscopy. Strains used are MYA-3404 (WT), HMY207 (*pmr1*Δ), HMY208 (*pmr1*Δ + *PMR1*), HMY181 (*och1*Δ), and HMY205 (*och1*Δ + *OCH1*). Scale bar = 15 μm. The experiments were performed in the absence of chitinase that disrupts cells aggregates in the null mutant strains.


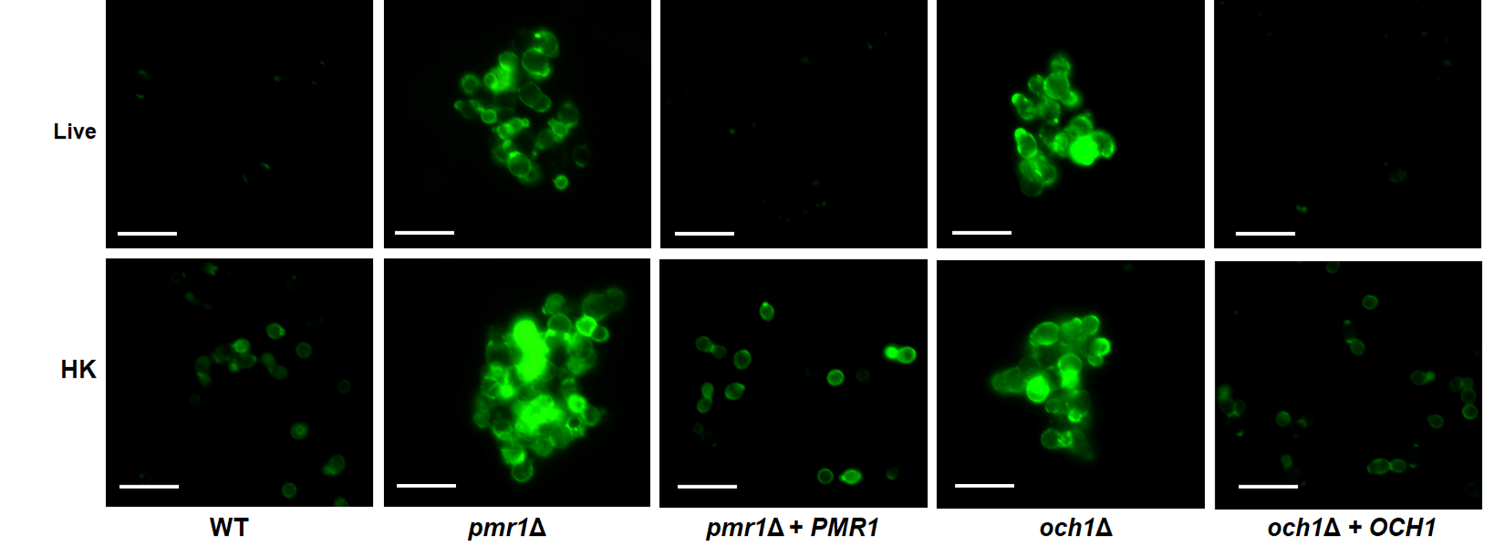


**Figure S2: The cell-wall structural polysaccharide chitin is significantly exposed at the cell surface of the *C. tropicalis* *pmr1*Δ and *och1*Δ null mutants.** Live or heat-killed (HK) cells were incubated with fluorescein isothiocyanate-wheat germ agglutinin conjugate as described in the Experimental procedures and inspected under fluorescence microscopy. Strains used are MYA-3404 (WT), HMY207 (*pmr1*Δ), HMY208 (*pmr1*Δ + *PMR1*), HMY181 (*och1*Δ), and HMY205 (*och1*Δ + *OCH1*). Scale bar = 15 μm. The experiments were performed in the absence of chitinase that disrupts cells aggregates in the null mutant strains.


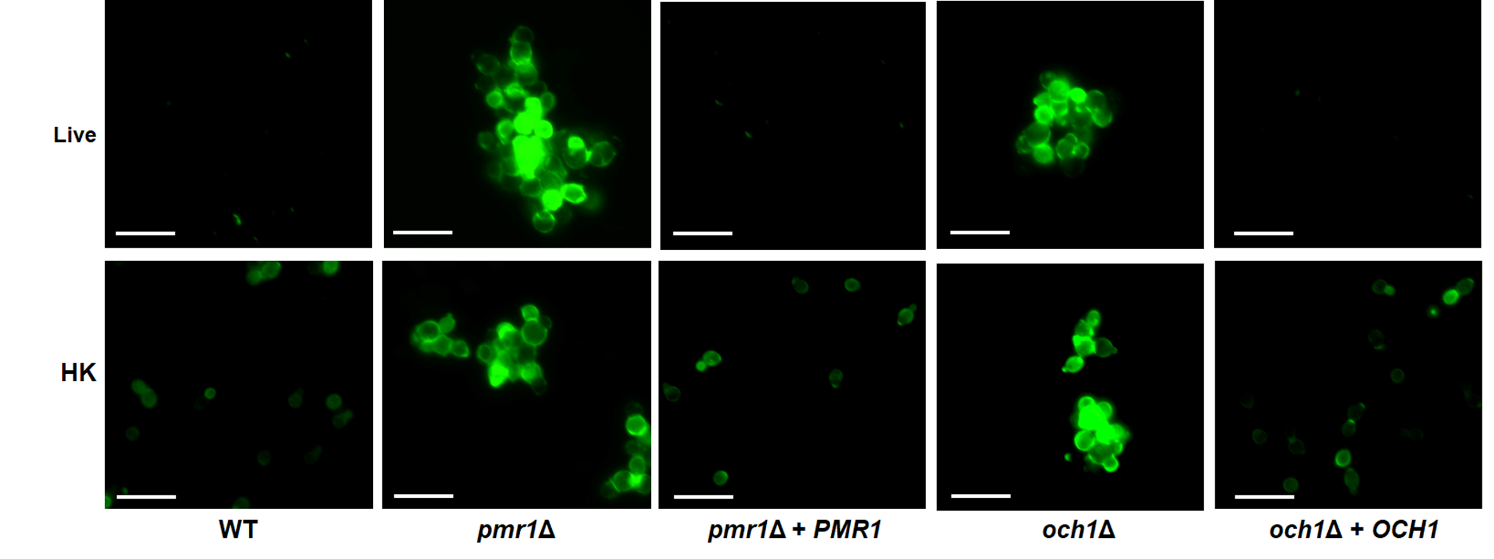


**Figure S3: The cell-wall structural polysaccharide β1,3-glucan is significantly exposed at the cell surface of the *C. tropicalis* *pmr1*Δ and *och1*Δ null mutants.** Live or heat-killed (HK) cells were incubated with the IgG Fc-Dectin-1 chimera as described in the Experimental procedures and inspected under fluorescence microscopy. Strains used are MYA-3404 (WT), HMY207 (*pmr1*Δ), HMY208 (*pmr1*Δ + *PMR1*), HMY181 (*och1*Δ), and HMY205 (*och1*Δ + *OCH1*). Scale bar = 15 μm. The experiments were performed in the absence of chitinase that disrupts cells aggregates in the null mutant strains.


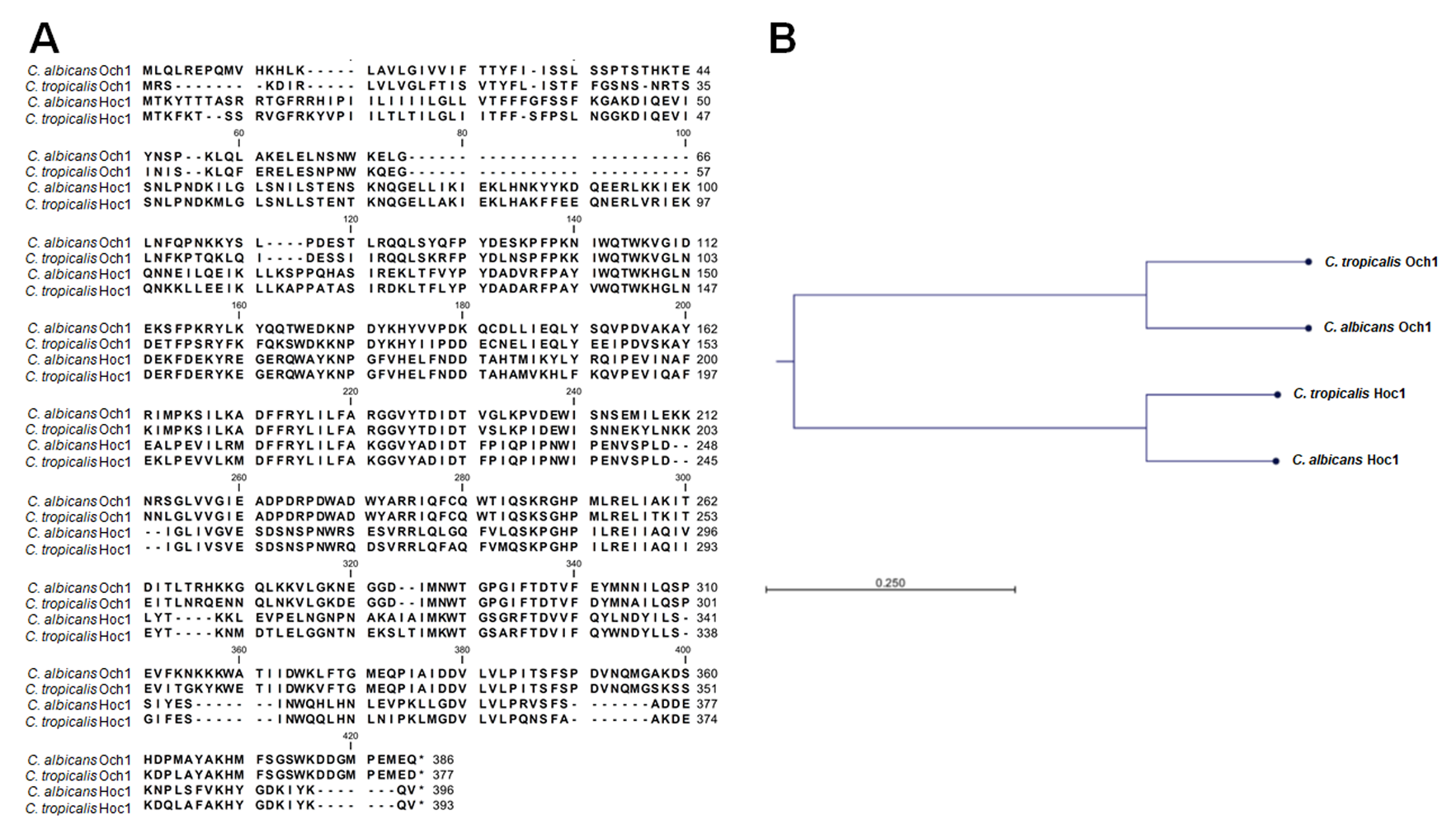


**Figure S4: Multiple sequence alignment and dendrogram of *C. albicans* and *C. tropicalis* Och1 and Hoc1.** Both the alignment (A) and the dendrogram (B) were generated using the CLC Genomics Workbench 12.0 (Qiagen) and the following sequences retrieved from the NCBI database: *C. albicans* Och1 (Accession code XP_716632), *C. albicans* Hoc1 (Accession code XP_716752), *C. tropicalis* Och1 (Accession code XP_002547957), and *C. tropicalis* Hoc1 (Accession code XP_002548535). The dendrogram was generated using the Neighbour-joining tree without a distance correction algorithm.
